# Association of Body Mass Index with Ischemic and Hemorrhagic Stroke

**DOI:** 10.3390/nu13072343

**Published:** 2021-07-09

**Authors:** Masahiro Shiozawa, Hidehiro Kaneko, Hidetaka Itoh, Kojiro Morita, Akira Okada, Satoshi Matsuoka, Hiroyuki Kiriyama, Tatsuya Kamon, Katsuhito Fujiu, Nobuaki Michihata, Taisuke Jo, Norifumi Takeda, Hiroyuki Morita, Sunao Nakamura, Koichi Node, Hideo Yasunaga, Issei Komuro

**Affiliations:** 1 The Department of Cardiovascular Medicine, The University of Tokyo, Tokyo 113-8655, Japan; m.palesaxeblue3205@gmail.com (M.S.); hitoh.ggl@gmail.com (H.I.); s-matsuoka@shin-tokyohospital.or.jp (S.M.); kiriyaman0427@gmail.com (H.K.); kamont-int@h.u-tokyo.ac.jp (T.K.); fujiu-tky@g.ecc.u-tokyo.ac.jp (K.F.); norifutakeda@gmail.com (N.T.); hiroymorita@gmail.com (H.M.); komuro_tky2000@yahoo.co.jp (I.K.); 2 The Department of Advanced Cardiology, The University of Tokyo, Tokyo 113-8655, Japan; 3 Global Nursing Research Center, Graduate School of Medicine, The University of Tokyo, Tokyo 113-8655, Japan; kojiromorita7@gmail.com; 4 Department of Prevention of Diabetes and Lifestyle-Related Diseases, Graduate School of Medicine, The University of Tokyo, Tokyo 113-8655, Japan; aokada@m.u-tokyo.ac.jp; 5 The Department of Cardiology, New Tokyo Hospital, Matsudo 270-2232, Japan; boss0606@pluto.plala.or.jp; 6 The Department of Health Services Research, The University of Tokyo, Tokyo 113-0033, Japan; gha10771@gmail.com (N.M.); jo.taisuke@gmail.com (T.J.); 7 Department of Cardiovascular Medicine, Saga University, Saga 849-8501, Japan; node@cc.saga-u.ac.jp; 8 The Department of Clinical Epidemiology and Health Economics, School of Public Health, The University of Tokyo, Tokyo 113-0033, Japan; yasunagah@m.u-tokyo.ac.jp

**Keywords:** body mass index, obesity, underweight, ischemic stroke, hemorrhagic stroke

## Abstract

Data on the association between body mass index (BMI) and stroke are scarce. We aimed to examine the association between BMI and incident stroke (ischemic or hemorrhagic) and to clarify the relationship between underweight, overweight, and obesity and stroke risk stratified by sex. We analyzed the JMDC Claims Database between January 2005 and April 2020 including 2,740,778 healthy individuals (Median (interquartile) age, 45 (38–53) years; 56.2% men; median (interquartile) BMI, 22.3 (20.2–24.8) kg/m^2^). None of the participants had a history of cardiovascular disease. Each participant was categorized as underweight (BMI <18.5 kg/m^2^), normal weight (BMI 18.5–24.9 kg/m^2^), overweight (BMI 25.0–29.9 kg/m^2^), or obese (BMI ≥ 30 kg/m^2^). We investigated the association of BMI with incidence stroke in men and women using the Cox regression model. We used restricted cubic spline (RCS) functions to identify the association of BMI as a continuous parameter with incident stroke. The incidence (95% confidence interval) of total stroke, ischemic stroke, and hemorrhagic stroke was 32.5 (32.0–32.9), 28.1 (27.6–28.5), and 5.5 (5.3–5.7) per 10,000 person-years in men, whereas 25.7 (25.1–26.2), 22.5 (22.0–23.0), and 4.0 (3.8–4.2) per 10,000 person-years in women, respectively. Multivariable Cox regression analysis showed that overweight and obesity were associated with a higher incidence of total and ischemic stroke in both men and women. Underweight, overweight, and obesity were associated with a higher hemorrhagic stroke incidence in men, but not in women. Restricted cubic spline showed that the risk of ischemic stroke increased in a BMI dose-dependent manner in both men and women, whereas there was a U-shaped relationship between BMI and the hemorrhagic stroke risk in men. In conclusion, overweight and obesity were associated with a greater incidence of stroke and ischemic stroke in both men and women. Furthermore, underweight, overweight, and obesity were associated with a higher hemorrhagic stroke risk in men. Our results would help in the risk stratification of future stroke based on BMI.

## 1. Introduction

Stroke is a major cause of death and disability [1,2,3]. In the United States, the annual incidence of stroke is approximately 795,000, of which approximately 610,000 are first-ever stroke events, and 185,000 are recurrent stroke events [1]. In the European countries, there were 2.3 million new cases diagnosed with stroke and 20.4 million people living with stroke in 2017 [4]. Obesity is an important risk factor for cardiovascular disease (CVD) [5,6,7,8,9] and is reported to be associated with a greater incidence of stroke [10,11,12]. Conversely, underweight is also associated with a higher risk of several CVDs and adverse clinical outcomes [13,14,15]. However, the data on the risk of underweight with incident stroke are scarce. Moreover, stroke can be categorized into two types, ischemic stroke, and hemorrhagic stroke; additionally, the pathology of these two subtypes should be separately discussed. For example, several studies have shown that body mass index (BMI) could influence the risk of ischemic or hemorrhagic stroke differently [16,17]. However, the association of wide-range BMI (including both obesity and underweight) with incident ischemic or hemorrhagic stroke has not been fully elucidated [10,11,12,16,17]. Furthermore, the distribution of BMI is different between men and women; therefore, the relationship between BMI and the risk of stroke could differ by sex [10,12]. In this study, we sought to examine the relationship between BMI and incident ischemic or hemorrhagic stroke stratified by sex using a nationwide epidemiological database.

## 2. Methods

The data from the JMDC Claims Database are available for anyone who would purchase it from JMDC Inc. (JMDC Inc.; Tokyo, Japan), which is a healthcare venture company in Tokyo, Japan.

### 2.1. Study Population

**Figure 1:**
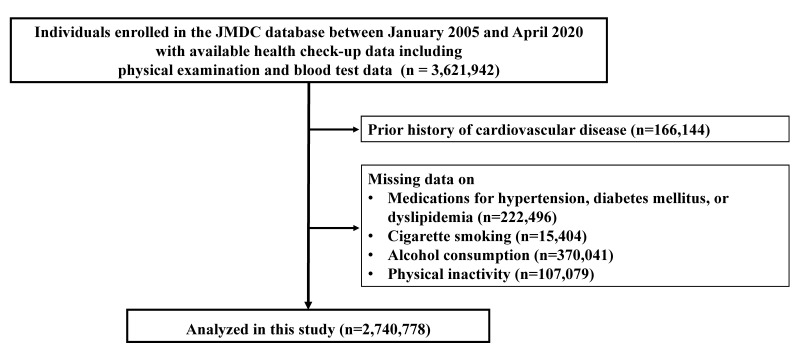
Flowchart. We extracted 3,621,942 individuals with available health check-up data including physical examination and blood test from the JMDC Claims Database between January 2005 and April 2020. We excluded individuals with CVD history of myocardial infarction, angina pectoris, stroke, heart failure, and atrial fibrillation or hemodialysis (n = 166,144), and those having missing data on medications for hypertension, diabetes mellitus, or dyslipidemia (n = 222,496), cigarette smoking (n = 15,404), alcohol consumption (n = 370,041), and physical inactivity (n = 107,079). Finally, we included 2,740,778 participants in this study.

We conducted this retrospective observational study using the JMDC Claims Database between January 2005 and April 2020 [18,19,20,21,22,23]. The JMDC Claims Database includes the health insurance claims data from more than 60 insurers. The majority of insured individuals enrolled in the JMDC Claims Database are employees of relatively large companies. The JMDC Claims Database includes the individuals’ health check-up data, including demographics, prior medical history, medication status, and hospital claims recorded using the International Classification of Diseases, 10th Revision (ICD-10) coding. JMDC which is a healthcare venture company, collected the data on health check-up and clinical outcome such as diagnosis of stroke using ICD-10 code from insurer or medical institutes regularly, and assembled a database. We extracted 3,621,942 individuals with available health check-up data on BMI (12.5–60 kg/m^2^), blood pressure, and blood test results at health check-up from the JMDC Claims Database between January 2005 and April 2020. Subsequently, we excluded the individuals with a history of myocardial infarction, angina pectoris, stroke, heart failure, and atrial fibrillation or hemodialysis (n = 166,144), and those with missing data on medications for hypertension, diabetes mellitus, or dyslipidemia (n = 222,496), cigarette smoking (n = 15,404), alcohol consumption (n = 370,041), and physical inactivity (n = 107,079). Finally, 2,740,778 participants were included in this study (Figure 1).

### 2.2. Ethics

This study was conducted according to the ethical guidelines of our institution (approval by the Ethical Committee of The University of Tokyo: 2018–10862) and in accordance with the principles of the Declaration of Helsinki. The requirement for informed consent was waived because all the data from the JMDC Claims Database were de-identified.

### 2.3. Category of Body Mass Index

We categorized the study participants into four groups: underweight, normal weight, overweight, and obesity defined as BMI <18.5 kg/m^2^, 18.5–24.9 kg/m^2^, 25.0–29.9 kg/m^2^ and ≥30 kg/m^2^, respectively [14].

### 2.4. Measurements and Definitions

The data, including BMI, history of hypertension, diabetes mellitus, dyslipidemia, CVD, blood pressure, and fasting laboratory values were collected using standardized protocols at the health check-up. The information on cigarette smoking (current or non-current) and alcohol consumption (every day or not every day) were self-reported. Hypertension was defined as systolic blood pressure ≥ 140 mmHg, diastolic blood pressure ≥ 90 mmHg, or the use of blood pressure-lowering medications. Diabetes mellitus was defined as fasting glucose level ≥ 126 mg/dL or the use of glucose-lowering medications. Dyslipidemia was defined as low-density lipoprotein cholesterol level ≥ 140 mg/dL, high-density lipoprotein cholesterol level <40 mg/dL, triglyceride level ≥ 150 mg/dL, or the use of lipid-lowering medications. Physical inactivity was defined as not engaging in at least 30 min of exercise two or more times a week or not walking ≥ 1 h per day, as previously described [24]. 

### 2.5. Outcomes

The outcomes were collected between January 2005 and April 2020. The primary outcome was stroke (ICD-10: I630, I631, I632, I633, I634, I635, I636, I638, I639, I600, I601, I602, I603, I604, I605, I606, I607, I608, I609, I610, I611, I613, I614, I615, I616, I619, I629, and G459). We defined ischemic stroke as I630, I631, I632, I633, I634, I635, I636, I638, I639, and G459, and hemorrhagic stroke as I600, I601, I602, I603, I604, I605. I606, I607, I608, I609, I610, I611, I613, I614, I615, I616, I619, and I629.

### 2.6. Statistical Analysis

We analyzed the study population stratified by sex. The data are expressed as median (interquartile range) for continuous variables or number (percentage) for categorical variables. The summary statistics for the characteristics of participants between the four categories based on BMI were calculated. The statistical significance of differences among the four categories was determined using analysis of variance for continuous variables and chi-squared tests for categorical variables. We conducted Cox regression analyses to identify the association between BMI categories and incident stroke. The hazard ratios (HRs) were calculated in an unadjusted model (Model 1), an age-adjusted model (Model 2), and after adjustment for age, hypertension, diabetes mellitus, dyslipidemia, cigarette smoking, alcohol consumption, and physical inactivity (Model 3). We performed three sensitivity analyses. First, we analyzed the relationship between BMI as a continuous variable and incident stroke. To detect any possible linear or non-linear dependency in regression models and to allow for a flexible interpretation of the relationship between BMI as continuous data and stroke events, continuous changes in BMI were assessed through shape-restricted cubic spline (RCS) regression models. We put three cut-off points for BMI (18.5, 25.0, and 30.0 kg/m^2^) as the knots. HRs and 95% confidence interval (CI) for incident stroke were calculated for each value of BMI with respect to the reference BMI value of 23.0 kg/m^2^. Second, we used multiple imputation for missing data, as previously described. [18,25] On the assumption of data missing at random, we imputed the missing data for covariates using the chained equation method with 20 iterations as described by Aloisio [26]. The HRs and standard errors were obtained using Rubin’s rules [27]. Third, we analyzed the population after excluding hypertensive participants. The statistical significance was set at *p* < 0.05. The statistical analyses were performed using SPSS software (version 25, SPSS Inc., Chicago, IL, USA) and STATA (version 17; StataCorp LLC, College Station, TX, USA). 

## 3. Results

### 3.1. Baseline Clinical Characteristics

The baseline clinical characteristics are shown in Table 1. Overall, the median (interquartile range) age was 45 (38–53) years, and 1,538,982 participants (56.2%) were men. The median (interquartile range) BMI was 23.2 (21.3–25.5) kg/m^2^ in men and 21.0 (19.2–23.4) kg/m^2^ in women. The prevalence of hypertension, diabetes mellitus, and dyslipidemia increased with BMI in both men and women.

### 3.2. Body Mass Index Category and Stroke 

In men, during a mean follow-up of 1269 ± 928 days, 17,221 total strokes, 14,901 ischemic strokes, and 2,943 hemorrhagic strokes occurred. The incidence (95% confidence interval) of total stroke, ischemic stroke, and hemorrhagic stroke was 32.5 (32.0–32.9), 28.1 (27.6–28.5), and 5.5 (5.3–5.7) per 10,000 person-years in men. In women, during a mean follow-up of 1091 ± 893 days, 9159 total strokes, 8041 ischemic strokes, and 1443 hemorrhagic strokes occurred. The incidence (95% confidence interval) of total stroke, ischemic stroke, and hemorrhagic stroke was 25.7 (25.1–26.2), 22.5 (22.0–23.0), and 4.0 (3.8–4.2) per 10,000 person-years. Compared with the normal weight group, the incidence of total stroke and ischemic stroke was lower in the underweight group, whereas it was higher in the overweight and obese groups in both men and women. Compared with the normal weight group, the incidence of hemorrhagic stroke was lower in the underweight group, and higher in the overweight and obese groups in women. However, the incidence of the hemorrhagic group was higher in not only the overweight and obese groups, but also in the underweight group compared with the normal weight group in men. Multivariable Cox regression analyses showed that, compared with the normal weight group, overweight (HR 1.07, 95% CI 1.03–1.10) and obesity (HR 1.18, 95% CI 1.10–1.26) were associated with a higher incidence of total stroke in men. In women, compared with the normal weight group, overweight (HR 1.07, 95% CI 1.01–1.13) and obesity (HR 1.15, 95% CI 1.03–1.27) were also associated with a higher incidence of total stroke. In terms of ischemic stroke, overweight (HR 1.06, 95% CI 1.03–1.11) and obesity (HR 1.14, 95% CI 1.06–1.23) were associated with a higher risk than normal weight in men. Obesity was associated with a higher risk than normal weight in women (HR 1.13, 95% CI, 1.01–1.27). Notably, overweight, obesity, and underweight were not associated with the risk of hemorrhagic stroke in women. In men, overweight (HR 1.10, 95% CI 1.01–1.19) and obesity (HR 1.37, 95% CI 1.19–1.58) were associated with a greater risk of hemorrhagic stroke than normal weight. Furthermore, underweight was also associated with a higher risk (HR 1.58, 95% CI 1.30–1.91) (Table 2).

### 3.3. Restricted Cubic Spline 

**Figure 2:**
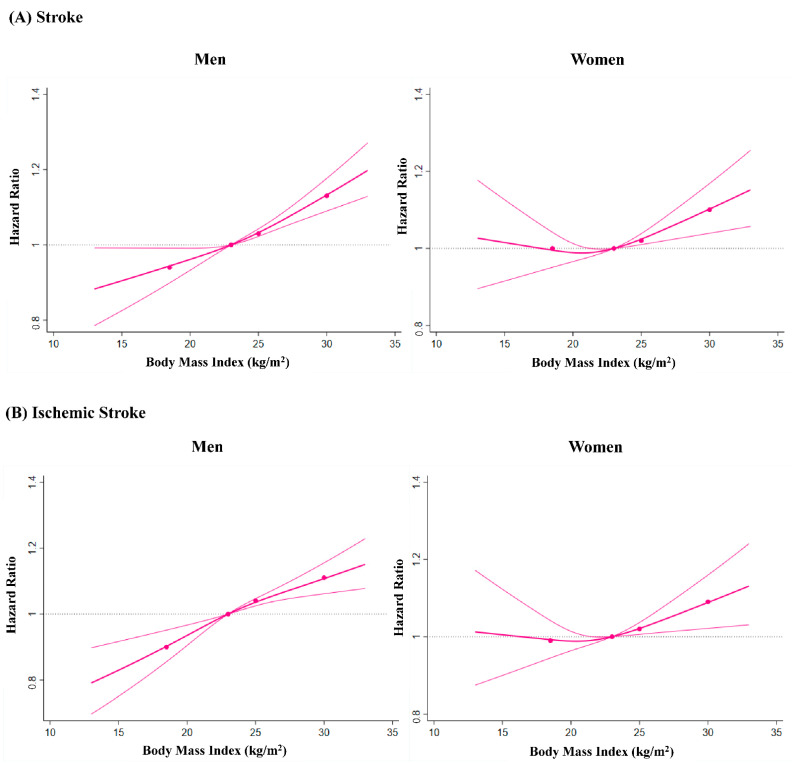

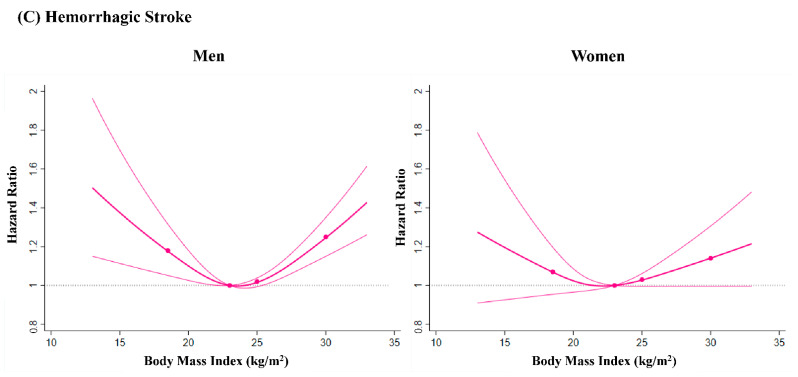
Restricted Cubic Spline. Restricted cubic spline of body mass index for total stroke (**A**), ischemic stroke (**B**), and hemorrhagic stroke (**C**).

Figure 2 shows the dose–response relationship between BMI and the risk of incident stroke. The association between BMI and the incidence of stroke was modeled using multivariable-adjusted spline regression models with a reference point set at BMI of 23 kg/m^2^. A linear dose–response relationship was observed between BMI and the risk of total stroke in men (Figure 2A). In women, RCS showed that the risk of total stroke was lowest at around 20 kg/m^2^ and increased in a dose-dependent manner after the BMI exceeded 20 kg/m^2^ (Figure 2A). There was a linear dose–response relationship between BMI and the risk of ischemic stroke in men (Figure 2B). In women, RCS showed that the incidence of ischemic stroke was lowest at around 20 kg/m^2^ and increased linearly after BMI exceeded approximately 20 kg/m^2^ (Figure 2B). There was a U-shaped relationship between BMI and the risk of hemorrhagic stroke with the bottoms of splines around 23–24 kg/m^2^ in men (Figure 2C). A dose-dependent association between BMI and the risk of hemorrhagic stroke was not evident in women (Figure 2C).

### 3.4. Multiple Imputation for Missing Data

We analyzed 3,455,798 participants (1,996,118 men and 1,459,680 women) after multiple imputations for missing data. Among these participants, 22,444 and 11,273 total stroke events occurred in men and women, respectively. In this model, overweight and obesity were associated with a higher incidence of total stroke and ischemic stroke in both men and women. Both obesity and underweight were associated with a greater risk of hemorrhagic stroke in men, but not in women (Table 3).

### 3.5. Non-Hypertensive Participants

After excluding hypertensive participants, we analyzed 1,199,658 men and 1,060,380 women in this model. Among them, 9310 and 6619 total stroke events occurred in men and women, respectively. Overweight and obesity were associated with a higher risk of total stroke or ischemic stroke in both men and women. Overweight and underweight were associated with a greater risk of hemorrhagic stroke in men. Notably, overweight, obesity, and underweight were not associated with a risk of hemorrhagic stroke in women (Table 4).

## 4. Discussion

The current analyses using a nationwide epidemiological database including approximately 2,700,000 people without a prevalent history of CVD, demonstrated that overweight and obesity were associated with a greater risk of total stroke and ischemic stroke in both men and women. Furthermore, underweight was associated with a greater incidence of hemorrhagic stroke in men, but not in women. These results did not change after multiple imputations for missing data or excluding hypertensive participants.

Various studies have been conducted to explore the relationship between BMI and future stroke events [10,11,12]. Prospective studies including approximately 900,000 people showed that the mortality due to stroke increased in a dose-dependent manner with baseline BMI after it exceeded 25 kg/m^2^ [28]. A population-based case–control study including 1,201 patients with ischemic stroke and 1154 controls aged 15–49 years showed that obesity defined as BMI > 30 kg/m^2^ was associated with an increased risk (odds ratio, 1.57; 95% CI, 1.28–1.94) [29]. Additionally, a higher BMI in adolescents was associated with a greater risk of ischemic stroke [30]. The analysis of the Atherosclerosis Risk in Communities (ARIC) Study including approximately 13,000 black and white people showed that obesity was associated with a greater risk of ischemic stroke irrespective of race [31]. Ischemic stroke was a major subtype of total stroke [1,32], and the majority of the studies focused on the relationship between BMI and ischemic stroke. However, there have been several studies on the association between BMI and hemorrhagic stroke. A recent analysis of the China National Stroke Screening and Intervention Program showed that obesity was associated with a higher risk of total and ischemic stroke, whereas underweight was associated with an elevated risk of hemorrhagic stroke [16]. An analysis of 234,863 Korean men aged 40–64 years reported a positive association between BMI and incident ischemic stroke, whereas a J-shaped association was observed between BMI and hemorrhagic stroke [17]. 

Our results were generally in line with previous studies, as described above. The present study had several strengths. First, this study included a large number of participants without a prior history of CVD. Additionally, the JMDC Claims Database included the medical claims records from employees’ insurance programs. Therefore, as long as each individual remained under coverage of the same insurance, the JMDC Claims Database could track the individuals’ clinical information, including the diagnosis of stroke events, even if the individual visits different medical institutions. Second, sex differences are important in the risk stratification and prevention of CVD, including stroke. Furthermore, the value of BMI is different between sexes; therefore, we separately analyzed men and women. The positive association of overweight/obesity with the incidence of total stroke and ischemic stroke was consistent in both men and women. However, underweight was associated with a higher incidence of hemorrhagic stroke only in men. Therefore, there could be a gender difference in the relationship between BMI and incident stroke, particularly hemorrhagic stroke. Although similar findings were reported in a previous study including Korean men [17], data including men in the United States did not show an increase in the risk of hemorrhagic stroke in individuals having lower BMI [10]. A previous study including 39,053 women in the United States examined the relationship between BMI and incident stroke and showed that BMI was a risk factor for total or ischemic stroke but not for hemorrhagic stroke, and this relationship was attenuated after adjustment for hypertension, diabetes mellitus, and hypercholesterolemia [12]. Compared with this study including women in the United States, the relationship between BMI and incident stroke (particularly ischemic stroke) was seemingly more obvious even after adjustment for covariates in women of this study. Therefore, further investigations are required to verify our results. However, these associations in men and women did not change after multiple imputations for missing data. Furthermore, because hypertension is known to be a strong risk factor for both ischemic and hemorrhagic stroke, we conducted a sensitivity analysis after excluding hypertensive participants. Even in this model, the main results did not change. Third, because the association between BMI and incident stroke could change depending on the cut-off value of BMI for underweight, overweight, and obesity, we conducted the RCS of BMI for incident stroke to deal with BMI as a continuous value. Similar to the association of overweight, obesity, and underweight with the risk of stroke, RCS demonstrated a dose-dependent increase in the risk of total stroke and ischemic stroke with BMI in men and women, and a U-shaped relationship between BMI and future hemorrhagic stroke risk in men. These results suggest a potential difference in the association of BMI with risk of future events between ischemic and hemorrhagic stroke, particularly in men.

This study has several limitations. Due to the nature of retrospective observational studies, our study could not conclude a causal relationship between baseline BMI and incident stroke. For example, our study showed that overweight and obesity were associated with an elevated risk of ischemic stroke. However, whether body weight loss could reduce the future risk of ischemic stroke in overweight or obese participants could not be discussed in this study. Similarly, although underweight was associated with a greater incidence of hemorrhagic stroke in men, the underlying mechanism for this association and the optimal management strategy for this population should be elucidated in future studies. For example, malnutrition and specific comorbidities may contribute to the elevated incidence of hemorrhagic stroke in underweight participants. However, the JMDC Claims Database does not include sufficient data to consider this point. Although the incidence of CVD in this database is acceptable compared with other epidemiological data in Japan, the recorded diagnoses of administrative databases are generally considered less well-validated. Since the JMDC Claims Database primarily included an employed population of working age, a selection bias (e.g., healthy worker bias) might exist. Therefore, further investigations are needed to determine whether our findings can be expanded to other populations of different races, ethnicities, and socioeconomic status. The main results did not change after multiple imputations for missing data. However, the substantial proportion of missing data should be considered a major study limitation. Although we used BMI in this study, dual energy X-ray absorptiometry is a standard method to evaluate a body composition including fat. This discrepancy might have contributed to the wide confidence intervals at high and low BMI levels on the RCS curve in women. Data on medication status were limited in this study. For example, use of antithrombotic medication or statin could influence the results. However, we were unable to analyze these data. Although the change in mediation status could also influence the results, data on the change in medication status were not available in this study. 

In conclusion, we analyzed a nationwide epidemiological database including a general population of 2,740,778 individuals with no prevalent history of CVD and found that overweight and obesity were associated with a higher incidence of total stroke and ischemic stroke in both sexes. Underweight was associated with a greater risk of future hemorrhagic stroke events in men, but not in women. Similarly, RCS showed that the risk of ischemic stroke dose-dependently increased with BMI in men and women, whereas there was a U-shaped relationship between BMI and future hemorrhagic stroke risk in men. Our results suggest that the association of BMI with subsequent risk differs between ischemic and hemorrhagic stroke, particularly in men.

## Figures and Tables

**Table 1:** Clinical Characteristics of Study Population.

	Men					Women				
	Body Mass Index Category					Body Mass Index Category				
	Normal -Weight (n = 1,013,302	Under -Weight (n = 61,704)	Over -Weight (n = 382,425)	Obesity (n = 81,551)	*p*-Value	Normal -Weight (n = 832,491)	Under -Weight (n = 180,421)	Over -Weight (n = 146,243)	Obesity (n = 42,641)	*p*-Value
Body Mass Index, kg/m^2^	22.2 (20.9–23.5)	17.7 (17.1–18.1)	26.5 (25.7–27.8)	31.9 (30.8–33.9)	<0.001	21.0 (19.7–22.5)	17.7 (17.0–18.1)	26.6 (25.7–27.9)	32.2 (30.9–34.4)	<0.001
Age	45 (38–53)	40 (28–49)	46 (40–54)	44 (38–50)	<0.001	44 (38–52)	42 (35–50)	47 (41–55)	45 (40–52)	<0.001
Hypertension	168,808 (16.7)	4403 (7.1)	125,180 (32.7)	40,933 (50.2)	<0.001	78,824 (9.5)	8078 (4.5)	36,944 (25.3)	17,570 (41.2)	<0.001
Diabetes Mellitus	38,041 (3.8)	1321 (2.1)	34,687 (9.1)	15,508 (19.0)	<0.001	10,050 (1.2)	984 (0.5)	8101 (5.5)	5345 (12.5)	<0.001
Dyslipidemia	402,085 (39.7)	8569 (13.9)	249,118 (65.1)	59,961 (73.5)	<0.001	218,254 (26.2)	24,740 (13.7)	73,805 (50.5)	25,321 (59.4)	<0.001
Cigarette Smoking	358,087 (35.3)	26,057 (42.2)	138,997 (36.3)	30,566 (37.5)	<0.001	89,573 (10.8)	21,531 (11.9)	18,698 (12.8)	6501 (15.2)	<0.001
Alcohol Drinking	334,709 (33.0)	15,635 (25.3)	111,393 (29.1)	13,905 (17.1)	<0.001	106,545 (12.8)	21,594 (12.0)	14,257 (9.7)	2680 (6.3)	<0.001
Physical Inactivity	511,731 (50.5)	31,675 (51.3)	208,506 (54.5)	47,022 (57.7)	<0.001	438,299 (52.6)	96,282 (53.4)	82,529 (56.4)	25,767 (60.4)	<0.001
SBP, mmHg	119 (110–128)	112 (104–122)	126 (117–135)	131 (123–141)	<0.001	111 (102–122)	106 (98–116)	122 (112–133)	129 (120–140)	<0.001
DBP, mmHg	74 (67–81)	69 (62–76)	80 (72–86)	83 (76–90)	<0.001	68 (61–76)	65 (59–72)	75 (68–83)	80 (72–88)	<0.001
Glucose, mg/dL	92 (87–99)	89 (84–95)	96 (89–105)	99 (91–113)	<0.001	89 (84–94)	86 (82–92)	93 (87–100)	97 (90–107)	<0.001
LDL-C/ mg/dL	119 (99–140)	98 (82–117)	130 (110–151)	132 (112–153)	<0.001	113 (94–135)	102 (86–122)	129 (108–151)	132 (113–154)	<0.001
HDL-C, mg/dL	59 (50–69)	66 (57–77)	51 (44–59)	47 (41–54)	<0.001	71 (61–81)	76 (67–87)	61 (52–71)	55 (48–64)	<0.001
Triglycerides, mg/dL	88 (63–127)	64 (48–85)	125 (88–181)	141 (101–202)	<0.001	63 (48–87)	55 (43–71)	91 (66–128)	107 (79–149)	<0.001

Data are reported as medians (interquartile range) and proportions (percentage). *p* values were calculated using chi-square tests for categorical variables and the analysis of variance for continuous variables. Participants were categorized into four groups based on body mass index (BMI); normal weight (BMI 18.5–24.9 kg/m^2^), underweight (BMI < 18.5 kg/m^2^), overweight (BMI 25.0–29.9 kg/m^2^), and obesity (BMI ≥ 30.0 kg/m^2^). SBP; systolic blood pressure, DBP; diastolic blood pressure, LDL-C; low-density lipoprotein cholesterol, HDL-C; high-density lipoprotein cholesterol.

**Table 2:** Association between Body Mass Index Category and Stroke Events Stratified by Sex.

	Men				Women			
	Normal Weight (n = 1,013,302	Underweight (n = 61,704)	Overweight (n = 382,425)	Obesity (n = 81,551)	Normal Weight (n = 832,491)	Underweight (n = 180,421)	Overweight (n = 146,243)	Obesity (n = 42,641)
Total Stroke								
No. of events	10,608	455	5089	1069	6197	1108	1443	411
Incidence	29.9 (29.4–30.5)	24.2 (22.1–26.5)	38.7 (37.6–39.8)	41.5 (39.0–44.0)	24.8 (24.2–25.5)	20.3 (19.2–21.6)	34.7 (32.9–36.5)	35.8 (32.5–39.4)
Model 1	1 (Reference)	0.81 (0.74–0.89)	1.30 (1.25–1.34)	1.39 (1.31–1.48)	1 (Reference)	0.82 (0.77–0.87)	1.40 (1.32–1.48)	1.44 (1.31–1.59)
Model 2	1 (Reference)	0.97 (0.89–1.07)	1.25 (1.21–1.29)	1.67 (1.57–1.78)	1 (Reference)	0.97 (0.91–1.04)	1.20 (1.13–1.27)	1.45 (1.31–1.60)
Model 3	1 (Reference)	1.05 (0.95–1.15)	1.07 (1.03–1.10)	1.18 (1.10–1.26)	1 (Reference)	1.02 (0.95–1.08)	1.07 (1.01–1.13)	1.15 (1.03–1.27)
Ischemic Stroke								
No. of events	9274	359	4395	873	5457	978	1257	349
Incidence	26.1 (25.6–26.7)	19.1 (17.2–21.1)	33.4 (32.4–34.4)	33.8 (31.6–36.1)	21.9 (21.3–22.4)	18.0 (16.9–19.1)	30.2 (28.6–31.9)	30.4 (27.4–33.7)
Model 1	1 (Reference)	0.73 (0.66–0.81)	1.28 (1.23–1.33)	1.30 (1.21–1.40)	1 (Reference)	0.82 (0.77–0.88)	1.38 (1.30–1.47)	1.39 (1.25–1.55)
Model 2	1 (Reference)	0.88 (0.79–0.98)	1.24 (1.19–1.28)	1.58 (1.47–1.69)	1 (Reference)	0.98 (0.91–1.05)	1.18 (1.11–1.26)	1.40 (1.25–1.55)
Model 3	1 (Reference)	0.95 (0.85–1.05)	1.06 (1.03–1.11)	1.14 (1.06–1.23)	1 (Reference)	1.02 (0.96–1.10)	1.06 (1.00–1.13)	1.13 (1.01–1.27)
Hemorrhagic Stroke								
No. of events	1699	111	891	242	953	175	239	76
Incidence	4.8 (4.5–5.0)	5.9 (4.9–7.1)	6.7 (6.3–7.2)	9.3 (8.2–10.6)	3.8 (3.6–4.1)	3.2 (2.8–3.7)	5.7 (5.0–6.5)	6.6 (5.3–8.2)
Model 1	1 (Reference)	1.24 (1.02–1.50)	1.42 (1.30–1.53)	1.97 (1.72–2.26)	1 (Reference)	0.84 (0.72–0.99)	1.51 (1.31–1.74)	1.74 (1.38–2.20)
Model 2	1 (Reference)	1.45 (1.20–1.76)	1.36 (1.26–1.48)	2.21 (1.93–2.53)	1 (Reference)	0.96 (0.82–1.13)	1.33 (1.16–1.54)	1.72 (1.37–2.18)
Model 3	1 (Reference)	1.58 (1.30–1.91)	1.10 (1.01–1.19)	1.37 (1.19–1.58)	1 (Reference)	1.02 (0.87–1.20)	1.09 (0.94–1.26)	1.14 (0.89–1.45)

The incidence rate was per 10,000 person-years. Model 1 = Unadjusted, Model 2 = Adjusted for age, Model 3 = Adjusted for age, hypertension, diabetes mellitus, dyslipidemia, cigarette smoking, alcohol consumption, and physical inactivity.

**Table 3:** Association between Body Mass Index Category and Stroke Events Stratified by Sex after Multiple Imputation for Missing Data.

	Men				Women			
	Normal Weight (n = 1,321,093)	Underweight (n = 83,425)	Overweight (n = 487,325)	Obesity (n = 104,275)	Normal Weight (n = 1,012,062)	Underweight (n = 220,072)	Overweight (n = 175,854)	Obesity (n = 51,692)
Total Stroke								
No. of events	13,772	612	6672	1388	7602	1375	1786	510
Incidence	28.4 (27.9–28.8)	22.4 (20.7–24.3)	37.9 (37.0–38.9)	40.0 (37.9–42.2)	24.5 (24.0–25.1)	20.2 (19.2–21.3)	34.8 (33.2–36.5)	35.6 (32.7–38.9)
Model 1	1 (Reference)	0.79 (0.73–0.86)	1.34 (1.30–1.38)	1.42 (1.34–1.50)	1 (Reference)	0.83 (0.78–0.87)	1.42 (1.35–1.50)	1.46 (1.33–1.59)
Model 2	1 (Reference)	0.99 (0.91–1.07)	1.26 (1.23–1.30)	1.66 (1.58–1.76)	1 (Reference)	0.98 (0.93–1.04)	1.21 (1.15–1.28)	1.44 (1.32–1.58)
Model 3	1 (Reference)	1.06 (0.98–1.15)	1.08 (1.05–1.11)	1.18 (1.12–1.25)	1 (Reference)	1.03 (0.97–1.09)	1.07 (1.02–1.13)	1.13 (1.03–1.24)
Ischemic Stroke								
No. of events	12,015	494	5787	1131	6690	1209	1554	437
Incidence	24.7 (24.3–25.2)	18.1 (16.6–19.8)	32.9 (32.0–33.7)	32.5 (30.7–34.5)	21.6 (21.1–22.1)	17.8 (16.8–18.8)	30.3 (28.8–31.8)	30.5 (27.8–33.5)
Model 1	1 (Reference)	0.73 (0.67–0.80)	1.33 (1.29–1.37)	1.32 (1.24–1.41)	1 (Reference)	0.82 (0.78–0.88)	1.40 (1.33–1.48)	1.42 (1.29–1.56)
Model 2	1 (Reference)	0.92 (0.84–1.00)	1.25 (1.22–1.29)	1.57 (1.48–1.67)	1 (Reference)	0.98 (0.93–1.05)	1.20 (1.13–1.26)	1.41 (1.28–1.55)
Model 3	1 (Reference)	0.98 (0.90–1.07)	1.08 (1.05–1.12)	1.14 (1.07–1.21)	1 (Reference)	1.03 (0.97–1.10)	1.07 (1.01–1.13)	1.13 (1.02–1.25)
Hemorrhagic Stroke								
No. of events	2253	139	1132	312	1189	220	301	91
Incidence	4.6 (4.4–4.8)	5.1 (4.3–6.0)	6.4 (6.0–6.8)	8.9 (8.0–10.0)	3.8 (3.6–4.0)	3.2 (2.8–3.7)	5.8 (5.2–6.5)	6.3 (5.1–7.8)
Model 1	1 (Reference)	1.10 (0.93–1.31)	1.39 (1.29–1.49)	1.95 (1.73–2.19)	1 (Reference)	0.84 (0.73–0.98)	1.53 (1.35–1.74)	1.66 (1.34–2.06)
Model 2	1 (Reference)	1.33 (1.12–1.58)	1.31 (1.22–1.41)	2.17 (1.92–2.44)	1 (Reference)	0.97 (0.84–1.12)	1.35 (1.19–1.53)	1.64 (1.32–2.02)
Model 3	1 (Reference)	1.44 (1.21–1.71)	1.06 (0.98–1.14)	1.37 (1.21–1.55)	1 (Reference)	1.02 (0.89–1.18)	1.09 (0.96–1.25)	1.06 (0.85–1.33)

The incidence rate was per 10,000 person-years. Model 1 = Unadjusted, Model 2 = Adjusted for age, Model 3 = Adjusted for age, hypertension, diabetes mellitus, dyslipidemia, cigarette smoking, alcohol consumption, and physical inactivity.

**Table 4:** Association between Body Mass Index Category and Stroke Events Stratified by Sex in Non-Hypertensive Participants.

	Men				Women			
	Normal Weight (n = 844,494)	Underweight (n = 57,301)	Overweight (n = 257,245)	Obesity (n = 40,618)	Normal Weight (n = 753,667)	Underweight (n = 172,343)	Overweight (n = 109,299)	Obesity (n = 25,071)
Total Stroke								
No. of events	6508	320	2203	279	4730	943	793	153
Incidence	22.0 (21.5–22.6)	18.3 (16.4–20.5)	24.9 (23.8–25.9)	22.1 (19.7–24.9)	20.8 (20.2–21.4)	18.1 (16.9–19.2)	25.3 (23.6–27.1)	22.9 (19.5–26.8)
Model 1	1 (Reference)	0.84 (0.75–0.94)	1.13 (1.08–1.19)	1.02 (0.90–1.15)	1 (Reference)	0.87 (0.81–0.93)	1.22 (1.13–1.31)	1.10 (0.94–1.30)
Model 2	1 (Reference)	0.97 (0.87–1.09)	1.14 (1.09–1.20)	1.32 (1.17–1.49)	1 (Reference)	0.99 (0.92–1.06)	1.13 (1.05–1.22)	1.26 (1.07–1.48)
Model 3	1 (Reference)	0.99 (0.88–1.10)	1.09 (1.04–1.15)	1.20 (1.06–1.35)	1 (Reference)	1.00 (0.93–1.08)	1.09 (1.01–1.18)	1.20 (1.02–1.41)
Ischemic Stroke								
No. of events	5785	253	1958	249	4222	840	709	142
Incidence	19.6 (19.1–20.1)	14.5 (12.8–16.4)	22.1 (21.1–23.1)	19.8 (17.5–22.4)	18.6 (18.0–19.2)	16.1 (15.0–17.2)	22.6 (21.0–24.4)	21.2 (18.0–25.0)
Model 1	1 (Reference)	0.75 (0.66–0.85)	1.13 (1.08–1.19)	1.02 (0.90–1.16)	1 (Reference)	0.87 (0.80–0.93)	1.22 (1.13–1.32)	1.15 (0.97–1.36)
Model 2	1 (Reference)	0.87 (0.76–0.98)	1.15 (1.09–1.21)	1.34 (1.18–1.52)	1 (Reference)	0.99 (0.92–1.07)	1.13 (1.04–1.22)	1.31 (1.11–1.55)
Model 3	1 (Reference)	0.88 (0.77–1.00)	1.09 (1.04–1.15)	1.21 (1.07–1.38)	1 (Reference)	1.01 (0.94–1.09)	1.09 (1.00–1.18)	1.24 (1.05–1.47)
Hemorrhagic Stroke								
No. of events	902	78	316	37	643	135	110	12
Incidence	3.0 (2.8–3.2)	4.5 (3.6–5.6)	3.6 (3.2–4.0)	2.9 (2.1–4.0)	2.8 (2.6–3.0)	2.6 (2.2–3.1)	3.5 (2.9–4.2)	1.8 (1.0–3.1)
Model 1	1 (Reference)	1.48 (1.18–1.87)	1.17 (1.03–1.33)	0.98 (0.71–1.37)	1 (Reference)	0.91 (0.76–1.10)	1.25 (1.02–1.53)	0.64 (0.36–1.13)
Model 2	1 (Reference)	1.68 (1.34–2.12)	1.17 (1.03–1.33)	1.16 (0.84–1.61)	1 (Reference)	1.01 (0.84–1.21)	1.17 (0.96–1.43)	0.70 (0.39–1.23)
Model 3	1 (Reference)	1.66 (1.32–2.10)	1.15 (1.01–1.31)	1.12 (0.81–1.57)	1 (Reference)	0.99 (0.83–1.20)	1.18 (0.97–1.45)	0.71 (0.40–1.27)

The incidence rate was per 10,000 person-years. Model 1 = Unadjusted, Model 2 = Adjusted for age, Model 3 = Adjusted for age, diabetes mellitus, dyslipidemia, cigarette smoking, alcohol consumption, and physical inactivity.

## Data Availability

The data from the JMDC Claims Database are available for anyone who would purchase it from JMDC Inc. (JMDC Inc.; Tokyo, Japan; https://www.jmdc.co.jp/en/index), which is a healthcare venture company in Tokyo, Japan.

## Non-Standard Abbreviations and Acronyms

BMI	Body Mass Index
CI	Confidence Interval
CVD	Cardiovascular Disease
HR	Hazard Ratio
RCS	Restricted Cubic Spline

## References

[1] Virani S.S., Alonso A., Benjamin E.J., Bittencourt M.S., Callaway C.W., Carson A.P., Chamberlain A.M., Chang A.R., Cheng S., Delling F.N. (2020). Heart Disease and Stroke Statistics-2020 Update: A Report from the American Heart Association. Circulation.

[2] Feigin V.L., Nguyen G., Cercy K., Johnson C.O., Alam T., Parmar P.G., Abajobir A.A., Abate K.H., Abd-Allah F., Abejie A.N. (2018). Global, Regional, and Country-Specific Lifetime Risks of Stroke, 1990 and 2016. N. Engl. J. Med..

[3] Feigin V.L., Mensah G.A., Norrving B., Murray C.J., Roth G.A., GBD 2013 Stroke Panel Experts Group (2015). Atlas of the Global Burden of Stroke (1990–2013): The GBD 2013 Study. Neuroepidemiology.

[4] Timmis A., Townsend N., Gale C.P., Torbica A., Lettino M., Petersen S.E., Mossialos E.A., Maggioni A.P., Kazakiewicz D., May H.T. (2020). European Society of Cardiology: Cardiovascular Disease Statistics 2019. Eur. Heart J..

[5] Guh D.P., Zhang W., Bansback N., Amarsi Z., Birmingham C.L., Anis A.H. (2009). The incidence of co-morbidities related to obesity and overweight: A systematic review and meta-analysis. BMC Public Health.

[6] Hubert H.B., Feinleib M., McNamara P.M., Castelli W.P. (1983). Obesity as an independent risk factor for cardiovascular disease: A 26-year follow-up of participants in the Framingham Heart Study. Circulation.

[7] Poirier P., Giles T.D., Bray G.A., Hong Y., Stern J.S., Pi-Sunyer F.X., Eckel R.H. (2006). Obesity and cardiovascular disease: Pathophysiology, evaluation, and effect of weight loss: An update of the 1997 American Heart Association Scientific Statement on Obesity and Heart Disease from the Obesity Committee of the Council on Nutrition, Physical Activity, and Metabolism. Circulation.

[8] Yusuf S., Hawken S., Ounpuu S., Bautista L., Franzosi M.G., Commerford P., Lang C.C., Rumboldt Z., Onen C.L., Lisheng L. (2005). Obesity and the risk of myocardial infarction in 27,000 participants from 52 countries: A case-control study. Lancet.

[9] Calle E.E., Thun M.J., Petrelli J.M., Rodriguez C., Heath C.W. (1999). Body-mass index and mortality in a prospective cohort of U.S. adults. N. Engl. J. Med..

[10] Kurth T., Gaziano J.M., Berger K., Kase C.S., Rexrode K.M., Cook N.R., Buring J.E., Manson J.E. (2002). Body mass index and the risk of stroke in men. Arch Intern Med..

[11] Yatsuya H., Toyoshima H., Yamagishi K., Tamakoshi K., Taguri M., Harada A., Ohashi Y., Kita Y., Naito Y., Yamada M. (2010). Body mass index and risk of stroke and myocardial infarction in a relatively lean population: Meta-analysis of 16 Japanese cohorts using individual data. Circ. Cardiovasc. Qual. Outcomes.

[12] Kurth T., Gaziano J.M., Rexrode K.M., Kase C.S., Cook N.R., Manson J.E., Buring J.E. (2005). Prospective study of body mass index and risk of stroke in apparently healthy women. Circulation.

[13] Senoo K., Nakata M., Teramukai S., Kumagai M., Yamamoto T., Nishimura H., Lip G.Y.H., Matoba S. (2021). Relationship Between Body Mass Index and Incidence of Atrial Fibrillation in Young Japanese Men- The Nishimura Health Survey. Circ. J..

[14] Itoh H., Kaneko H., Kiriyama H., Kamon T., Fujiu K., Morita K., Yotsumoto H., Michihata N., Jo T., Takeda N. (2021). Reverse J-shaped relationship between body mass index and in-hospital mortality of patients hospitalized for heart failure in Japan. Heart Vessel..

[15] Echouffo-Tcheugui J.B., Masoudi F.A., Bao H., Curtis J.P., Heidenreich P.A., Fonarow G.C. (2019). Body mass index and outcomes of cardiac resynchronization with implantable cardioverter-defibrillator therapy in older patients with heart failure. Eur. J. Heart Fail..

[16] Qi W., Ma J., Guan T., Zhao D., Abu-Hanna A., Schut M., Chao B., Wang L., Liu Y. (2020). Risk Factors for Incident Stroke and Its Subtypes in China: A Prospective Study. J. Am. Heart Assoc..

[17] Song Y.M., Sung J., Davey Smith G., Ebrahim S. (2004). Body mass index and ischemic and hemorrhagic stroke: A prospective study in Korean men. Stroke.

[18] Kaneko H., Itoh H., Yotsumoto H., Kiriyama H., Kamon T., Fujiu K., Morita K., Michihata N., Jo T., Morita H. (2020). Association of body weight gain with subsequent cardiovascular event in non-obese general population without overt cardiovascular disease. Atherosclerosis.

[19] Goto A., Goto M., Terauchi Y., Yamaguchi N., Noda M. (2016). Association Between Severe Hypoglycemia and Cardiovascular Disease Risk in Japanese Patients with Type 2 Diabetes. J. Am. Heart Assoc..

[20] Wake M., Onishi Y., Guelfucci F., Oh A., Hiroi S., Shimasaki Y., Teramoto T. (2018). Treatment patterns in hyperlipidaemia patients based on administrative claim databases in Japan. Atherosclerosis.

[21] Kawasaki R., Konta T., Nishida K. (2018). Lipid-lowering medication is associated with decreased risk of diabetic retinopathy and the need for treatment in patients with type 2 diabetes: A real-world observational analysis of a health claims database. Diabetes Obes. Metab..

[22] Kaneko H., Itoh H., Kiriyama H., Kamon T., Fujiu K., Morita K., Michihata N., Jo T., Takeda N., Morita H. (2021). Lipid Profile and Subsequent Cardiovascular Disease among Young Adults Aged <50 Years. Am. J. Cardiol..

[23] Kaneko H., Itoh H., Yotsumoto H., Kiriyama H., Kamon T., Fujiu K., Morita K., Kashiwabara K., Michihata N., Jo T. (2020). Cardiovascular Health Metrics of 87,160 Couples: Analysis of a Nationwide Epidemiological Database. J. Atheroscler. Thromb..

[24] Kaneko H., Itoh H., Kamon T., Fujiu K., Morita K., Michihata N., Jo T., Morita H., Yasunaga H., Komuro I. (2020). Association of Cardiovascular Health Metrics with Subsequent Cardiovascular Disease in Young Adults. J. Am. Coll. Cardiol..

[25] Yagi M., Yasunaga H., Matsui H., Morita K., Fushimi K., Fujimoto M., Koyama T., Fujitani J. (2017). Impact of Rehabilitation on Outcomes in Patients with Ischemic Stroke: A Nationwide Retrospective Cohort Study in Japan. Stroke.

[26] Aloisio K.M., Swanson S.A., Micali N., Field A., Horton N.J. (2014). Analysis of partially observed clustered data using generalized estimating equations and multiple imputation. Stata J..

[27] Rubin D.B., Schenker N. (1991). Multiple imputation in health-care databases: An overview and some applications. Stat. Med..

[28] Whitlock G., Lewington S., Sherliker P., Clarke R., Emberson J., Halsey J., Qizilbash N., Collins R., Peto R., Prospective Studies Collaboration (2009). Body-mass index and cause-specific mortality in 900,000 adults: Collaborative analyses of 57 prospective studies. Lancet.

[29] Mitchell A.B., Cole J.W., McArdle P.F., Cheng Y.C., Ryan K.A., Sparks M.J., Mitchell B.D., Kittner S.J. (2015). Obesity increases risk of ischemic stroke in young adults. Stroke.

[30] Bardugo A., Fishman B., Libruder C., Tanne D., Ram A., Hershkovitz Y., Zucker I., Furer A., Gilon R., Chodick G. (2021). Body Mass Index in 1.9 Million Adolescents and Stroke in Young Adulthood. Stroke.

[31] Yatsuya H., Folsom A.R., Yamagishi K., North K.E., Brancati F.L., Stevens J., Atherosclerosis Risk in Communities Study Investigators (2010). Race- and sex-specific associations of obesity measures with ischemic stroke incidence in the Atherosclerosis Risk in Communities (ARIC) study. Stroke.

[32] Krishnamurthi R.V., Feigin V.L., Forouzanfar M.H., Mensah G.A., Connor M., Bennett D.A., Moran A.E., Sacco R.L., Anderson L.M., Truelsen T. (2013). Global and regional burden of first-ever ischaemic and haemorrhagic stroke during 1990–2010: Findings from the Global Burden of Disease Study 2010. Lancet Glob. Health.

